# Bioinformatic analysis of peripheral blood RNA-sequencing sensitively detects the cause of late graft loss following overt hyperglycemia in pig-to-nonhuman primate islet xenotransplantation

**DOI:** 10.1038/s41598-019-55417-y

**Published:** 2019-12-11

**Authors:** Hyun-Je Kim, Ji Hwan Moon, Hyunwoo Chung, Jun-Seop Shin, Bongi Kim, Jong-Min Kim, Jung-Sik Kim, Il-Hee Yoon, Byoung-Hoon Min, Seong-Jun Kang, Yong-Hee Kim, Kyuri Jo, Joungmin Choi, Heejoon Chae, Won-Woo Lee, Sun Kim, Chung-Gyu Park

**Affiliations:** 1 Xenotransplantation Research Center, Seoul National University College of Medicine, Seoul, 03080 Republic of Korea; 2 Department of Microbiology and Immunology, Seoul National University College of Medicine, Seoul, 03080 Republic of Korea; 3 Department of Biomedical Sciences, Seoul National University Graduate School, Seoul, 03080 Republic of Korea; 4 Interdisciplinary Program in Bioinformatics, Seoul National University, Seoul, 08826 Republic of Korea; 5 Department of Computer Engineering, Chungbuk National University, Cheongju, 28644 Republic of Korea; 6 Division of Computer Science, Sookmyung Women’s University, Seoul, 04310 Republic of Korea; 7 Bioinformatics Institute, Department of Computer Science and Engineering, Seoul National University, Seoul, 08826 Republic of Korea; 8 Department of Computer Science & Engineering, Seoul National University, Seoul, 08826 Republic of Korea; 9 Cancer Research Institute, Seoul National University College of Medicine, Seoul, 03080 Republic of Korea; 10 Institute of Endemic Diseases, Seoul National University College of Medicine, Seoul, 03080 Republic of Korea; 11 Biomedical Research Institute, Seoul National University Hospital, Seoul, 03080 Republic of Korea; 12 Present Address: Department of Dermatology and the Laboratory of Inflammatory Skin Diseases, Icahn School of Medicine at Mount Sinai, New York, NY 10029 USA; 13 Present Address: Department of Biological Sciences, University at Buffalo, Buffalo, NY 14260 USA

**Keywords:** Biochemical reaction networks, Diabetes

## Abstract

Clinical islet transplantation has recently been a promising treatment option for intractable type 1 diabetes patients. Although early graft loss has been well studied and controlled, the mechanisms of late graft loss largely remains obscure. Since long-term islet graft survival had not been achieved in islet xenotransplantation, it has been impossible to explore the mechanism of late islet graft loss. Fortunately, recent advances where consistent long-term survival (≥6 months) of adult porcine islet grafts was achieved in five independent, diabetic nonhuman primates (NHPs) enabled us to investigate on the late graft loss. Regardless of the conventional immune monitoring methods applied in the post-transplant period, the initiation of late graft loss could rarely be detected before the overt graft loss observed via uncontrolled blood glucose level. Thus, we retrospectively analyzed the gene expression profiles in 2 rhesus monkey recipients using peripheral blood RNA-sequencing (RNA-seq) data to find out the potential cause(s) of late graft loss. Bioinformatic analyses showed that highly relevant immunological pathways were activated in the animal which experienced late graft failure. Further connectivity analyses revealed that the activation of T cell signaling pathways was the most prominent, suggesting that T cell-mediated graft rejection could be the cause of the late-phase islet loss. Indeed, the porcine islets in the biopsied monkey liver samples were heavily infiltrated with CD3^+^ T cells. Furthermore, hypothesis test using a computational experiment reinforced our conclusion. Taken together, we suggest that bioinformatics analyses with peripheral blood RNA-seq could unveil the cause of insidious late islet graft loss.

## Introduction

The Edmonton protocol was introduced in 2000^1^, and since then human pancreatic islet transplantation has become an established treatment option for type 1 diabetic patients who frequently experienced fatal hypoglycemic unawareness^2^. However, over half of the patients transplanted with human islets returned to the insulin-dependent, diabetic state within 5 years^3,4^. The causes for this late islet graft loss are still controversial. Previous reports encompass a higher rate of islet apoptosis due to endoplasmic reticulum (ER) stress^5,6^, hypoxia^7,8^ in end-portal venules within the liver, and recurrent autoimmunity^9^. In addition, there were evidences of metabolic deterioration due to lipid accumulated around the islets (lipotoxicity)^10,11^ and toxicity of immunosuppressive drugs^12,13^, which could all result in graft loss. Last but not least, insufficient immunosuppression could also be an important cause of chronic islet loss especially due to antibody-mediated rejection processes^14^. However, none of the above could clearly and single-handedly explain the exact causes of chronic islet graft loss.

Recently, we reported consistent long-term (≥6 months) porcine islet graft survivals in five independent monkeys^15^. This unique opportunity allowed us to examine how the porcine islets are lost in the late phase of islet xenotransplantation. Here, we selected two monkeys with the same immunosuppressive regimen to analyze the cause of late graft loss in islet xenotransplantation: one (R051) had stable graft function for the entire follow-up periods and the other (R080) lost graft function around 160 days post-transplantation (DPT). Peripheral blood RNA-seq and subsequent bioinformatics analyses using Time-series RNA-seq analysis package (TRAP)^16^ hinted on the possibility of immune rejection in R080. Further *in silico* analyses focused on the interactions of graft loss period-related activated pathways (GLPAPs) proposed that lymphocytes- or platelet-mediated rejection might have been the cause of late graft loss.

## Results

### Peripheral blood RNA sequencing

**Figure 1:**
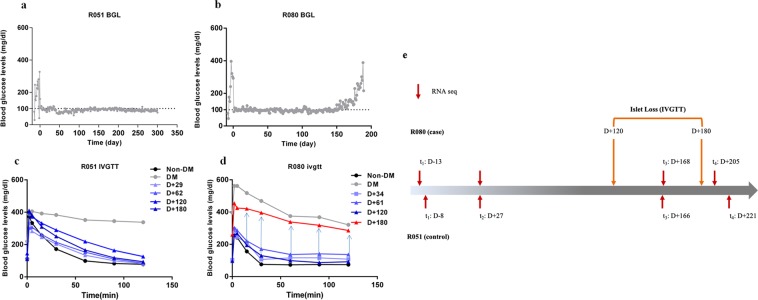
Graft function and experimental scheme. (**a**,**b**) Blood glucose levels of R051 and R080. R080 showed gradual increase of blood glucose level around DPT 150. (**c**,**d**) IVGTT results of R051 and R080. Between DPT 120 and 180, R080 showed prominent glucose intolerance. (**e**) Sampling time point for RNA-seq. Whole blood archives were used for RNA-seq. (t_1_: before transplantation, t_2_: one month after transplantation, t_3_: immediate after increase of blood glucose in R080 and corresponding time point for R051, t_4_: after overt hyperglycemia in R080 and corresponding time point for R051).

R051 had shown complete normoglycemia and glucose disposal capacity for the entire follow-up periods, whereas the other (R080) exhibited relatively early hyperglycemia around DPT160, suggesting a graft failure. Intravenous glucose tolerance test (IVGTT) had shown that the porcine islet graft loss in R080 was in progress between DPT120 and 180 (Fig. 1a~d, processed from published data^15^). However, vital signs and routine laboratory examinations including complete blood cell count (CBC), liver function test (LFT), C-reactive protein (CRP), kidney function test (blood urine nitrogen/creatinine), electrolyte panel (sodium, potassium and chloride), lipase and amylase had shown no abnormal findings in both of the monkeys (data not shown). Also, monitoring of peripheral blood lymphocyte subsets by flow cytometry and ELISPOT (Supplementary Fig. S1), and titer of donor-specific antibody by enzyme-linked immunosorbent assay (ELISA)^15^ had not revealed any marked changes. Since recent report showed gene expression perturbation in peripheral blood could reflect graft site event^17,18^, we performed RNA sequencing with the archives of whole blood samples taken at four different time points from graft-losing R080 *vs*. graft-stable R051 (Fig. 1e) to explore the cause of chronic islet loss happened in R080.

### Graft loss period-related activated pathways (GLPAPs) defined by TRAP

After confirming the validity of RNA-seq data, we used TRAP^16^ to determine which pathways had played roles in the graft loss process. Because t_2_ and t_3_ represent the graft-maintaining and the graft-losing period, respectively in R080, we focused on these time points and selected pathways as follows: i) select up-regulated pathways with p-value under 0.05 from the results yielded by TRAP comparing t_3_ and t_2_ in R080, ii) select up-regulated pathways with p-value under 0.05 from the results yielded by comparing R080 and R051 at t_3_ and then take the pathways belonging to the intersection of those sets (Supplementary Fig. S2). As a result, we could obtain 59 pathways among 287 pathways in Kyoto Encyclopedia of Genes and Genomes (KEGG)^19^ Rhesus database and these pathways were named GLPAPs as can be seen in Table 1.

**Table 1:** Graft losing period-related activated pathways (GLPAPs).

Pathway	Name	Category
mcc04062	Chemokine signaling pathway	Immune system
mcc04611	Platelet activation	
mcc04620	Toll-like receptor signaling pathway	
mcc04621	NOD-like receptor signaling pathway	
mcc04623	Cytosolic DNA-sensing pathway	
mcc04650	Natural killer cell mediated cytotoxicity	
mcc04660	T cell receptor signaling pathway	
mcc04662	B cell receptor signaling pathway	
mcc04664	Fc epsilon RI signaling pathway	
mcc04670	Leukocyte transendothelial migration	
mcc04010	MAPK signaling pathway	Signal transduction
mcc04012	ErbB signaling pathway	
mcc04022	cGMP-PKG signaling pathway	
mcc04064	NF-kappa B signaling pathway	
mcc04068	FoxO signaling pathway	
mcc04070	Phosphatidylinositol signaling system	
mcc04152	AMPK signaling pathway	
mcc04370	VEGF signaling pathway	
mcc04630	Jak-STAT signaling pathway	
mcc04668	TNF signaling pathway	
mcc04910	Insulin signaling pathway	Endocrine system
mcc04915	Estrogen signaling pathway	
mcc04917	Prolactin signaling pathway	
mcc04918	Thyroid hormone synthesis	
mcc04919	Thyroid hormone signaling pathway	
mcc04921	Oxytocin signaling pathway	
mcc03013	RNA transport	Translation
mcc04210	Apoptosis	Cell growth and death
mcc05211	Renal cell carcinoma	Cancers: Specific types
mcc05212	Pancreatic cancer	
mcc05213	Endometrial cancer	
mcc05214	Glioma	
mcc05215	Prostate cancer	
mcc05219	Bladder cancer	
mcc05220	Chronic myeloid leukemia	
mcc05221	Acute myeloid leukemia	
mcc05223	Non-small cell lung cancer	
mcc04141	Protein processing in endoplasmic reticulum	Folding, sorting and degradation
mcc04320	Dorso-ventral axis formation	Development
mcc04380	Osteoclast differentiation	
mcc04540	Gap junction	Cellular communication
mcc04810	Regulation of actin cytoskeleton	Cell motility
mcc04961	Endocrine and other factor-regulated calcium reabsorption	Excretory system
mcc04722	Neurotrophin signaling pathway	Nervous system
mcc04725	Cholinergic synapse	
mcc04060	Cytokine-cytokine receptor interaction	Signaling molecules and interaction
mcc05142	Chagas disease (American trypanosomiasis)	Infectious diseases: Parasitic
mcc05143	African trypanosomiasis	
mcc05144	Malaria	
mcc05161	Hepatitis B	Infectious diseases: Viral
mcc05162	Measles	
mcc05164	Influenza A	
mcc05166	HTLV-I infection	
mcc05168	Herpes simplex infection	
mcc05169	Epstein-Barr virus infection	
mcc04970	Salivary secretion	Digestive system
mcc05200	Pathways in cancer	Cancers: Overview
mcc05203	Viral carcinogenesis	
mcc05205	Proteoglycans in cancer	

59 out of 287 pathways in Rhesus KEGG database were selected after applying of TRAP algorithm.

After obtaining 59 of GLPAPs, we were able to calculate p-values for each ‘category’ of the pathways using Fisher’s exact test to determine how significantly GLPAPs were enriched in each category. To calculate p-values, we constructed a contingency table with two variables: GLPAP and category. Each cell of the table was filled by the number of the pathways according to the standard if the pathway belongs to the category or not and if the pathway is GLPAP or not (Supplementary Fig. S3). The p-values for each category are shown in Table 2. The most enriched category was found to be ‘immune system,’ although we had not found significant perturbation of immunological parameters in routine immune monitoring system. This finding strongly implied that immunological responses were somehow activated and ongoing during t_3_ in R080 compared to t_2_ in R080 and corresponding time points in R051.

**Table 2:** Significantly enriched categories of GLPAPs.

Category	P-value
Immune system	0.0001962
Cancers: Specific types	0.0003236
Infectious diseases: Viral	0.0003591
Signal transduction	0.0120207
Infectious diseases: Parasitic	0.1036661
Endocrine system	0.1076348
Development	0.1083940
Cell motility	0.2055749
Cancers: Overview	0.2132333
Digestive system	0.6910951
Nervous system	1.0000000
Cell growth and death	1.0000000
Cellular communication	1.0000000
Excretory system	1.0000000
Folding, sorting and degradation	1.0000000
Signaling molecules and interaction	1.0000000
Translation	1.0000000

Categories are listed in ascending order of p-values calculated by Fisher’s exact test. ‘Immune system’ category pathways were highly enriched.

### Pathway interaction network analisys

**Figure 2:**
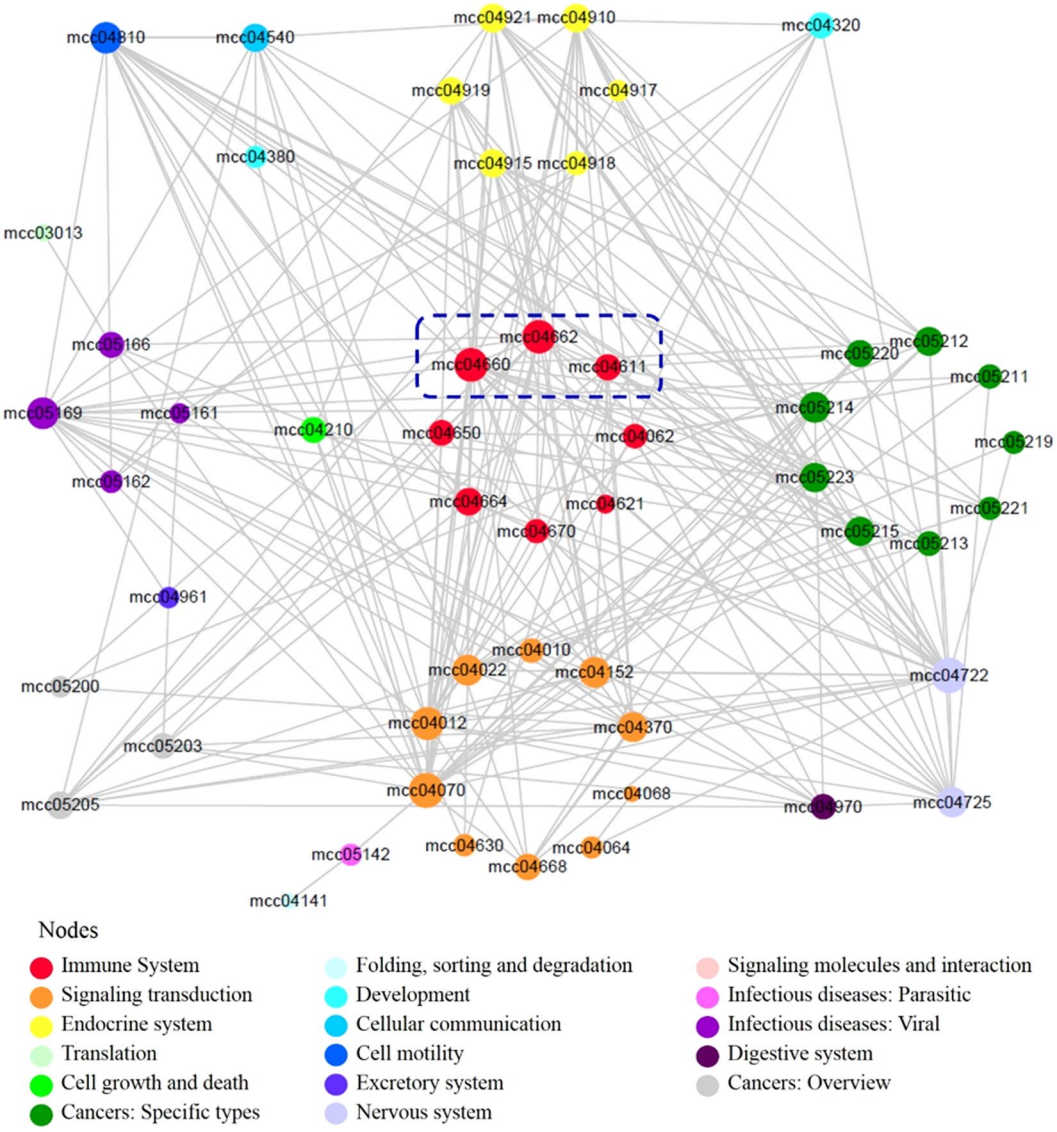
Pathway interaction network. Blue dotted rectangle represents T cell receptor signaling pathway (mcc04660), B cell receptor signaling pathway (mcc04662), and Platelet activation (mcc04611). The size of the nodes reflects the closeness centrality of each node. The network was visualized by Cytoscape^40^.

Even though we found that the pathways categorized as ‘immune system’ were enriched mostly after GLPAP filtering, we were not able to specify pathways which had been potentially responsible for late graft loss. Because biological pathways usually function in a cooperative manner by constituting a network, understanding the network of pathways can provide the insight about which pathways are important in a given condition. Therefore, it would be desirable to analyze the network of the pathways to find out the most interacting pathways to induce the late graft loss among GLPAPs. To this end, we constructed a pathway interaction network of GLPAPs using PINTnet^20^. There were 52 pathways out of 59 GLPAPs connected by 225 edges in the network (Fig. 2). We calculated closeness centrality for every node and used degree information to analyze which pathways had played a central role in the network to induce the biological response at t_3_ of R080. We focused only on the pathways belonging to the ‘immune system’ because ‘immune system’ was the most enriched category as mentioned in the previous section. The pathways with the closeness centrality value and degree higher than the average closeness centrality value and the average degree of all the nodes in the network were considered meaningful. Among eight pathways of ‘immune system,’ three met the criteria and these pathways were T cell receptor signaling pathway, B cell receptor signaling pathway, and platelet activation. The pathways are shown in Table 3.

**Table 3:** The closeness centrality and the degree of GLPAPs in immune system.

ID	Name	Closeness centrality	Degree
mcc04660	T cell receptor signaling pathway	0.6	21
mcc04662	B cell receptor signaling pathway	0.59302326	20
mcc04611	Platelet activation	0.46788991	11
mcc04664	Fc epsilon RI signaling pathway	0.49514563	8
mcc04650	Natural killer cell mediated cytotoxicity	0.45945946	7
mcc04062	Chemokine signaling pathway	0.43589744	7
mcc04670	Leukocyte transendothelial migration	0.43220339	5
mcc04621	NOD-like receptor signaling pathway	0.33774834	1

The average closeness centrality and the average degree of all GLPAPs in the network were 0.4669 and 8.65 respectively and the values were used as the cutoff values to determine if a GLPAP was meaningful in the pathway interaction network. Only T cell receptor signaling pathway, B cell receptor signaling pathway, and Platelet activation satisfied the cutoff values. The pathways are highlighted by underlines.

### T cell-mediated immune rejection confirmed by biopsy

**Figure 3:**
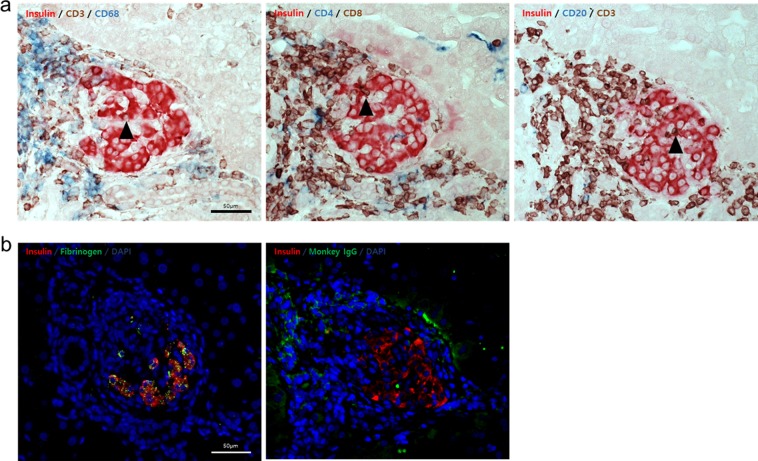
Histology of islet xenografts on DPT184. (**a**) The islet graft was heavily infiltrated by several types of immune cells in R080. Immune cells largely consisted of CD3^+^ T cells. Both CD4^+^ and CD8^+^ cells infiltrated the graft. CD68^+^ cells were also observed. Black arrowheads indicate intra-graft infiltrating T cells. The scale bar applies universally. (**b**) Immunofluorescence staining against insulin (red), fibrinogen, and monkey IgG (left and right, respectively; both green). Blue denotes nuclei stained by DAPI. The scale bar applies universally.

The results from bioinformatics analyses on RNA-seq suggested that T cell-mediated immune rejection toward the porcine islets had been in progress at t_3_ of R080. We wanted to confirm whether our analysis reflected real biological processes. Thus, we collected liver biopsy samples at DPT184 from R080 from the archives and examined graft histology by immunohistochemistry. Indeed, we found that insulin-positive islet grafts were heavily infiltrated by mostly CD3^+^ T cells (Fig. 3a). Moreover, immunofluorescence staining also showed that porcine islet grafts had been positive for fibrinogen and monkey IgG (Fig. 3b), the result which was in parallel with the pathway interaction network analysis. Because we did not find any noticeable change in peripheral blood immune cell phenotyping, antibody titers, ELISPOT analysis, and other routine laboratory tests, we concluded that bioinformatics analyses on peripheral blood RNA-seq could only gave us information on whether immunological reaction in response to the graft was in progress or not and furthermore, which biological pathways would be activated during this process in the transplant recipient.

### Hypothesis evaluation using network propagation

To reconfirm our findings, we sought to computationally test each hypothesis which could explain the islet graft loss. We selected five hypotheses that are known to cause the late graft loss^14^. Those were ER stress^5,21–23^, islet exhaustion^24^, lipotoxicity^10,25–27^, chronic graft rejection^28,29^, and toxicity of immunosuppressants^12^. To evaluate the five hypotheses, we designed and performed a computational experiment: the rationale behind the experiment was that if a hypothesis had been the cause of the late graft loss and the genes related to the hypothesis had been important, the global effects of the genes of the hypothesis should have been similar to the gene expression profile that we measured. To measure the global effects of the genes, we used the network propagation algorithm^30^. The evaluation process was as follows: The five hypotheses-related seed genes were collected, and a differentially expressed gene (DEG) profile was established. Then, a protein-protein interaction (PPI) network was mapped with the seed genes and the global effects were measured using network propagation. After the genes were ranked under each hypothesis, Pearson’s correlation analysis was performed between the DEG profile ranks and the ranks calculated by the network propagation. This was to test which of the five hypotheses represented by the seed genes produced the gene expression profile similar to the DEG profile we measured. In other words, we tried to see how much the participation of genes in the actual biological process that had induced the graft loss coincided with the perturbation in the expression of genes in the given condition. Furthermore, we performed random simulations for 1000 times and calculated empirical p-values to test the significance of the coefficient as shown in Methods. The result is shown in Table 4 and we were able to see that the correlation coefficients of ‘chronic graft rejection’ was the highest and most significant. In addition, we carried out the same process for top 100 genes of network propagation results for each hypothesis. As shown in Table 5, chronic graft rejection was the highest in terms of the coefficient. This result suggested and supported that late-phase graft loss reflected by the condition-specific changes in gene expression of R080 was explained the best by chronic graft rejection.

**Table 4:** Ranking comparison between network propagation results and differential expression.

Hypothesis	Coefficient	p-value	Empirical p-value
ERstress	0.031115500	0.001246017	0.048
IsletExh	0.049513322	0.000048063	0.292
Lipotoxicity	0.051612597	0.000022611	0.251
CGR	0.087461960	0.000000000	0.010
ToxImmDrug	0.050939480	0.000028885	0.275

Pearson’s correlation coefficients of each hypothesis. IsletExh, CGR, and ToxImmDrug stand for islet exhaustion, chronic graft rejection, and toxicity of immunosuppressant, respectively. The coefficient of chronic graft rejection was the highest.

**Table 5:** Ranking comparison between network propagation results and differential expression.

Hypothesis	Coefficient	p-value
ERstress	0.235867693	0.018154182
IsletExh	0.154287565	0.125356981
Lipotoxicity	0.188772704	0.059979769
CGR	0.556480178	0.000000001
ToxImmDrug	0.536431327	0.000000008

Correlation coefficients of ranking comparison for the 100 most-influenced genes from the network propagation results. Chronic graft rejection showed the highest coefficient.

## Discussion

Pancreatic islet transplantation is currently one of the best treatment options for end-stage type 1 diabetes patients^31^. Although the engraftment of islets has been successful short-term, it relatively lacked long-term durability, resulting in late-phase graft failure in some islet transplant recipients^2^. Likewise, we and others have found that the porcine islet grafts were also lost in the transplant recipient monkeys within 6~30 months after transplantation in pig-to-NHP islet xenotransplantation^32^. Luckily, we were able to experience consistent, prolonged graft survival in rhesus monkeys^15^, and thus this retrospective analysis was performed in hopes to unearth the cause of late graft loss in pig-to-NHP islet xenotransplantation. To our knowledge, there have not been studies that were focused on the cause of late graft loss nor the mechanism of immune rejection in islet transplantation models of higher mammals.

Because we were not able to find any definitive explanation of islet loss with routine laboratory tests including biochemical and immunological assays in peripheral blood from R080, we used RNA-seq technology to quantify the amount of transcript in the samples obtained from whole blood taken at various time points after transplantation. Then, we carried out pathway analysis and pathway interaction network analysis based on TRAP and PINTnet, respectively. By performing pathway analyses, we found 59 activated pathways and named those GLPAPs (Table 1). Furthermore, we categorized GLPAPs to retrieve meaningful information. Indeed, mostly enriched category was revealed as ‘immune system’ (Table 2). This highly suggested that cause of graft loss in chronic phase in R080 is due to insufficient immune suppression, i.e. immune rejection. Subsequently, we constructed a pathway interaction network using GLPAPs as nodes to reveal which pathways played a central role in the given condition and found that ‘T cell receptor signaling pathway,’ ‘B cell signaling pathway,’ and ‘platelet activation’ were the most interconnected pathways (Fig. 2 & Table 3). This information suggested that our immunosuppressive regimen in the maintenance period should be revised and fortified to overcome the late graft loss.

Computationally, the above findings were reinforced by hypothesis testing. We can measure the influence of some nodes of interests to other nodes on a network using the network propagation. Likewise, we can measure the influence of genes on a biological network or a gene regulatory network. If we map genes on a curated biological network, select some genes as seed genes and run network propagation, we can measure the influence of the seed genes to other genes and rank the genes by the influence they received. Top 100 genes, for example, are the most influenced 100 genes. In our study, network propagation and subsequent correlation analysis revealed that chronic rejection-related genes had been the most related to the late graft loss (Table 4). It is noteworthy that our computational analyses are fairly relevant to the previous findings concerning the chronic rejection in transplantation. The B cells and antibodies have been known as the culprit of chronic rejection in solid organ transplantation^29^. There also have been reports indicative of platelet’s contribution in the chronic rejection of transplanted organs^33^. T cells have been mentioned very little regarding the chronic rejection of transplanted organs, but our results might have discovered that T cells may partake significantly in the chronic rejection of transplanted islets, at least in pig-to-NHP settings.

It would be of dire importance to show that the RNA-seq bioinformatics data truly represented the immunobiology of the recipient which experienced late graft loss. We retrieved the archives of liver biopsy materials of R080, and performed IHC that could prove the pertinence of the three GLPAPs (‘T cell receptor signaling pathway,’ ‘B cell signaling pathway,’ ‘platelet activation’) to the rejection. Accordingly, we could observe the infiltration of T cells to the graft site as well as antibody and fibrinogen accumulation (Fig. 3). Since these immune responses had not been conspicuous via routine immune monitoring (Supplementary Fig. S1) as mentioned before, our result calls for further studies on the monitoring of graft-proximal immunology in islet transplantation.

It is intriguing that only R080 experience immune system activation compared with R051 despite the usage of the same immunosuppressive regimen. To find out putative reason(s), we carefully reviewed pre-clinical symptoms, signs and laboratory data. Interestingly, we noticed that R080 had experienced two times of severe giardiasis-induced diarrhea around DPT90 and DPT120. This finding indicated that intestinal infections preceded the sampling for the RNA-seq about one month. There is increasing evidence that microbiota products could activate the innate immune system of the liver^34^, and it was reported by Xie *et al*. that microbiota alteration could result in acute rejection of rat liver allografts^35^. Because intestinal blood is drained to the liver via portal vein, we hypothesized that intestinal infection might have activated strong innate immunity and this in turn triggered adaptive immune response to the graft through heterologous immunity. In line with this notion, the immunologically hostile effects of infection on the transplanted allografts were reported^36^ and infection even might break down established tolerance to the graft in murine heart transplantation models^37^.

There are some limitations in our work. First, we only had one monkey which experienced relative early graft loss within the chronic phase after transplantation. Thus, our study cannot give a definite conclusion, but rather an intriguing insight to our field. We are planning to scale up our study using more animals to validate our bioinformatic analysis method. Second, although we suggested that three pathways might have been involved in immune rejection in the chronic phase, we only found the presence of the effectors in each pathway and could not present their actual involvement. Third, because rhesus pathways in KEGG were relatively insufficient, we were not able to analyze our data in high-resolution maps. For example, even though we were interested in CD40L or IL-6 signaling pathways in our setting, we were not able to analyze them because KEGG did not support those pathways. Lastly, we were not able to pinpoint single candidate molecule or a set of molecules which could be critically responsible for graft rejection. Our next works will focus on these questions.

## Methods

### Animals

Rhesus monkeys (*Macaca mulatta*), 3–4 years of age were used in our study. All animal experiment procedures were performed in compliance with the Guide for the Care and Use of Laboratory Animals prepared by the Institute of Laboratory Animal Resources and published by the National Institutes of Health (NIH Publication No. 86–23, revised 2011). The experiments were approved by Seoul National University (SNU) Institutional Animal Care and Use Committee (IACUC no. 15-0297-S1A0). Islet donor pigs, the Seoul National University (SNU) miniature pigs were bred as in designated pathogen-free (DPF) grade^38^.

### Porcine islet isolation transplantation into the monkey recipients

Adult porcine islet were isolated from pig and transplanted into the liver of rhesus monkey as described^15^. In brief, chemically diabetic induced rhesus monkeys were transplanted with porcine islet via jejunal vein after a laparotomy was performed.

### Immunosuppression

Induction immunosuppression included a regimen with anti-human CD154 monoclonal antibody, sirolimus (Rapamune®, Wyeth), and anti-thymocyte globulin (ATG, Thymoglobulin®, Genzyme). Sirolimus was administered daily to achieve stable trough levels between 3 and 8 ng/ml. CVF (100 U/kg, Quidel) was administered on day -1 of the transplant to prevent complement activation. TNF-α neutralizing monoclonal antibody, adalimumab (Humira^®^, Abbott Laboratories Ltd., Queenborough, UK) was administered subcutaneously 2~3 hrs before islet infusion with dose of 5 mg/kg. 10^6^ to 10^7^ *ex-vivo* expanded regulatory T cells were adoptively transferred after ATG depletion.

### IVGTT

After an overnight fasting without insulin, 0.5 g/kg of 50% dextrose solution with same volume of normal saline was infused i.v. for 1 min. Blood glucose levels were measured in monkeys before and 2, 5, 15, 30, 60, 90, and 120 min after infusion. Insulin and c-peptide levels were measured at the same time intervals as described previously^15^.

Hematology

### Enzyme-linked immunosorbent spot (ELISPOT) assay

ELISPOT analysis was performed by the method previously described^39^. 2.5 × 10^5^ monkey peripheral blood mononuclear cells were cultured with 5 × 10^5^ porcine splenocytes (30 Gy irradiation). The resulting spots were counted on a computer-assisted ELISpot Reader System (AID, Germany).

### Flow cytometry

Flow cytometry analyses of peripheral blood leukocytes were performed using the following monoclonal antibodies: CD3-FITC (FN-18; U-CyTech biosciences, Utrecht, The Netherlands), CD4-APC-Cy7 (OKT; BioLegend, San Diego, CA), CD8-PE-Cy7 (SK1; eBioscience, San Diego, CA), FoxP3-PE (PCH101; eBioscience), HLA-DR-PerCP-eFluor710 (L243; eBioscience), CD20-PE (2H7; Thermo Fisher Scientific, Waltham, MA), CD14-Alexa488 (M5E2; Biolegend), CD16-APC (3G8; BD Biosciences). Absolute counts of leukocytes were measured with 123count eBeadsTM (Thermo Fisher Scientific). FACSCanto II flow cytometer (BD Biosciences; San Jose, CA) and FACSDiva software (BD Biosciences) were used for analyses.

### RNA sequencing

Total RNA from peripheral blood of rhesus monkey was extracted using the Ambion Ribopure^TM^-Blood kit (Thermo Fisher Scientific) as recommended by the manufacturer. 500 μl of peripheral blood from the recipient monkeys was used for each sample. Eluted total RNA was stored in −80 °C. Next, RNA sequencing was performed. Purified total RNA was sequenced by Theragen Etex (Korea).

### Biopsy and immunohistochemistry

Biopsy and immunohistochemical staining was conducted as described previously^15^. Briefly, liver biopsy samples from the recipient monkeys were fixed in 10% neutral buffered formalin, and embedded in paraffin. Paraffin-embedded tissues were 4-μm sectioned using a microtome. Each de-paraffinized and hydrated section was incubated for 30 min with primary antibody cocktails for insulin (Santa Cruz Biotechnologies, Dallas, TX), CD3 (DAKO, Agilent, Santa Clara, CA), CD4 (Santa cruz Biotechnologies), CD8, CD20, and CD68 (all from Abcam, UK), and then washed four times in TBST. After staining procedure, all of the stained slides were dried at 60 °C, and mounted with aqueous mounting medium (Thermo Fisher Scientific). For immunofluorescence staining, anti-fibrinogen antibody and anti-insulin antibody (both from Abcam) were used as primary antibodies and, Alexa 488-conjugated anti-rabbit IgG and Alexa 647-conjugated anti-mouse IgG were used as secondary antibodies, respectively. FITC-conjugated anti-monkey IgG (Acris, Germany) was used to detect antibody involvement at the graft site. The stained sample was observed by Carl Zeiss Axio Imager A1 microscope and images were taken with a micrograph with AxioVision software (Carl Zeiss, Germany).

### Bioinformatic analyses

TRAP^16^ and pathway interaction network^20^ were performed as described previously. Network propagation was performed as follows: The genes related to the five hypotheses were collected as seed genes by the literature search and domain knowledge, and each hypothesis was represented by a set of genes. Next, DEG profile was established by measuring the expression change of each gene with calculating the log2 fold change between R080 and R051 at time point 3 and ranking the genes. At that time, we removed the genes of which the expression value was smaller than 1 in either R080 or R051 to prevent extremely high or low fold change yielded by the comparison between small numbers. Then, we mapped the seed genes on a PPI network. The number of nodes and edges in the network are 6,780 and 117,963 respectively. The number of seed genes are ten, nine, eight, ten, and nine for ER stress, islet exhaustion, lipotoxicity, chronic graft rejection, and toxicity of immunosuppressant, respectively. After that, the global effect of the seed genes was measured using network propagation and the genes in the PPI network were ranked for each hypothesis. Then, Pearson’s correlation coefficients were calculated between the ranking in the DEG profile and the ranking by the network propagation for each hypothesis. One thousand random simulations were run and empirical p-values were calculated to test the significance of the coefficient, as shown in the equation below:

$${p}^{i}=\frac{1}{N}\mathop{\sum }\limits_{j=1}^{N}\{\begin{array}{c}1\,if\,{c}_{ij} > {c}_{i}^{R}\\ 0\,otherwise\end{array}$$

where *i* indicates each hypothesis and it ranges from 1 to 5. *p*^*i*^ indicates the empirical p-value of *i*-th hypothesis. *N* is the number of random simulation and it is 1,000. *j* indicates the *j*-th random simulation. *c*_*ij*_ is the coefficient of *j*-th random simulation of *i*-th hypothesis. ${{\rm{c}}}_{i}^{R}$ is the reference coefficient of *i*-th hypothesis.

## Supplementary information

Supplementary Information
