# Supplementary material for: Bioinformatic analysis of peripheral blood RNA-sequencing sensitively detects the cause of late graft loss following overt hyperglycemia in pig-to-nonhuman primate islet xenotransplantation
Source: Sci Rep. 2019 Dec 11;9:18835. doi: 10.1038/s41598-019-55417-y (PMC6906328; doi:10.1038/s41598-019-55417-y)
Supplement: Supplementary file 1 — Supplementary Information [file 41598_2019_55417_MOESM1_ESM.pdf]

Bioinformatic analysis of peripheral blood RNA-sequencing sensitively detects the cause of late graft loss following overt hyperglycemia in pig-to-nonhuman primate islet xenotransplantation

Hyun-Je Kim[1,2,3,#,†], Ji Hwan Moon[4,#,‡], Hyunwoo Chung[1,2,3,#], Jun-Seop Shin[1], Bongi Kim[2], Jong-Min Kim[1], Jung-Sik Kim[1], Il-Hee Yoon[1], Byoung-Hoon Min[1], Seong-Jun Kang[1,2,3], Yong-Hee Kim[1], Kyuri Jo[5], Joungmin Choi[6], Heejoon Chae[6], Won-Woo Lee[1,2,3], Sun Kim[4,7,8,*], Chung-Gyu Park[1,2,3,9,10,11,*]

[1]Xenotransplantation Research Center, Seoul National University College of Medicine, Seoul, 03080, Republic of Korea

[2]Department of Microbiology and Immunology, Seoul National University College of Medicine, Seoul, 03080, Republic of Korea

[3]Department of Biomedical Sciences, Seoul National University Graduate School, Seoul, 03080, Republic of Korea

[4]Interdisciplinary Program in Bioinformatics, Seoul National University, Seoul, 08826, Republic of Korea

[5]Department of Computer Engineering, Chungbuk National University, Cheongju, 28644, Republic of Korea

[6]Division of Computer Science, Sookmyung Women's University, Seoul, 04310, Republic of Korea

[7]Bioinformatics Institute, Department of Computer Science and Engineering, Seoul National University, Seoul, 08826, Republic of Korea

[8]Department of Computer Science & Engineering, Seoul National University, Seoul, 08826, Republic of Korea

[9]Cancer Research Institute, Seoul National University College of Medicine, Seoul, 03080, Republic of Korea

[10]Institute of Endemic Diseases, Seoul National University College of Medicine, Seoul, 03080, Republic of Korea

[11]Biomedical Research Institute, Seoul National University Hospital, Seoul, 03080, Republic of Korea

[#] These authors contributed equally to this work.

[†] Hyun-Je Kim's current address is, Department of Dermatology and the Laboratory of Inflammatory Skin Diseases, Icahn School of Medicine at Mount Sinai, New York, NY 10029, USA.

[‡] Ji Hwan Moon's current address is, Department of Biological Sciences, University at Buffalo, Buffalo, NY 14260, USA.

* All correspondence should be addressed to Chung-Gyu Park, MD, PhD (chgpark@snu.ac.kr, chgpark@gmail.com) and Sun Kim, PhD (sunkim.bioinfo@snu.ac.kr)

Supplementary information

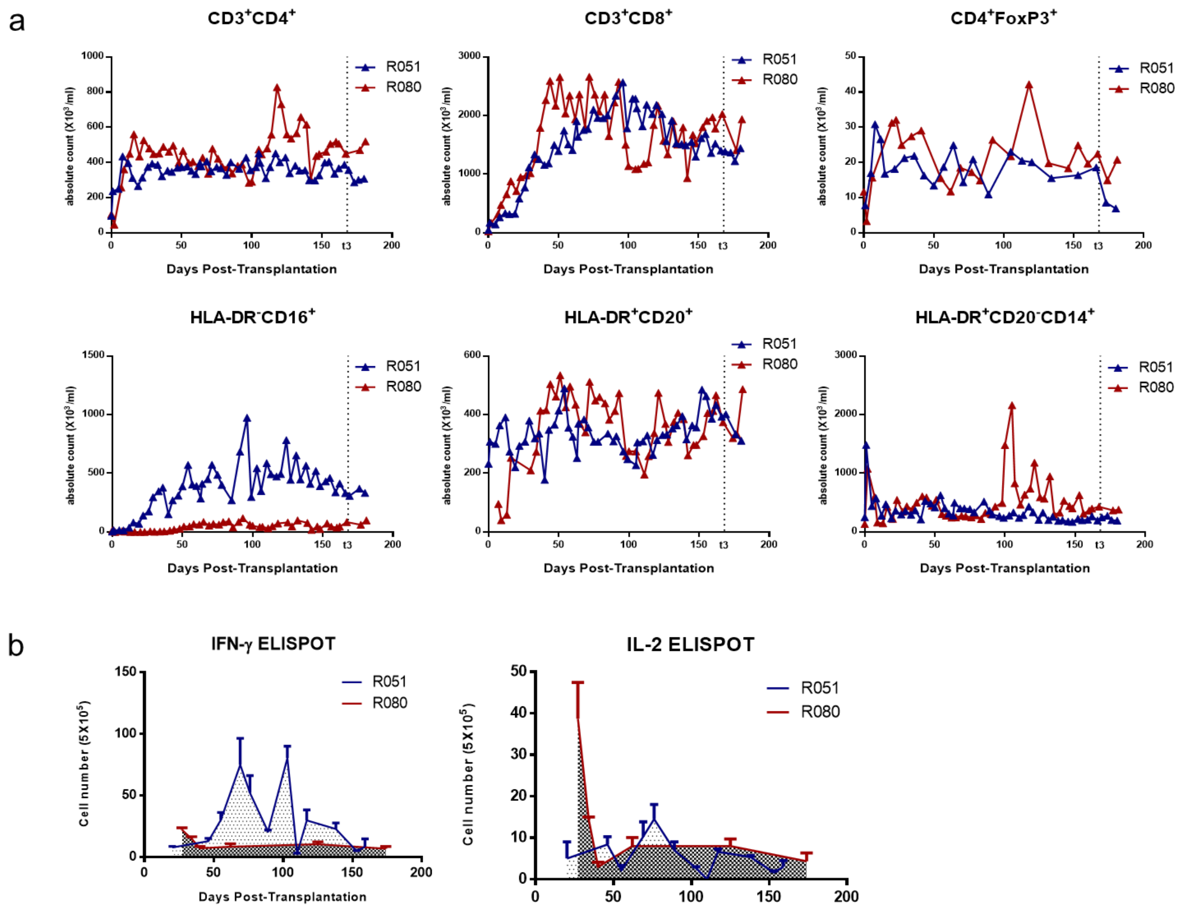

Supplementary Figure 1. Routine peripheral blood immune monitoring data of the two recipient monkeys. (**a**) Flow cytometry analysis of blood leukocyte subpopulations in the peripheral blood. The subsets were (from the top left panel, clockwise) CD4 T cells, CD8 T cells, regulatory T cells, monocytes, B cells, and NK cells. Dotted lines indicate t3 (DPT168). (**b**) ELISPOT analysis of cytokine-secreting cells from the peripheral blood in the two recipient monkeys.

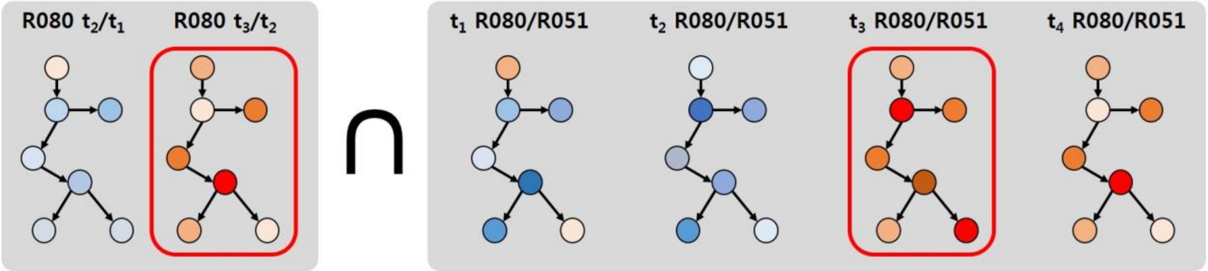

Supplementary Figure 2. Pathway filtering strategy. GLPAPs were selected by taking the

intersection of two TRAP results. One was to detect which pathways were activated at t_3 compared to t_2 in R080. The other was to detect which pathways were activated in R080 compared to R051 at t_3.

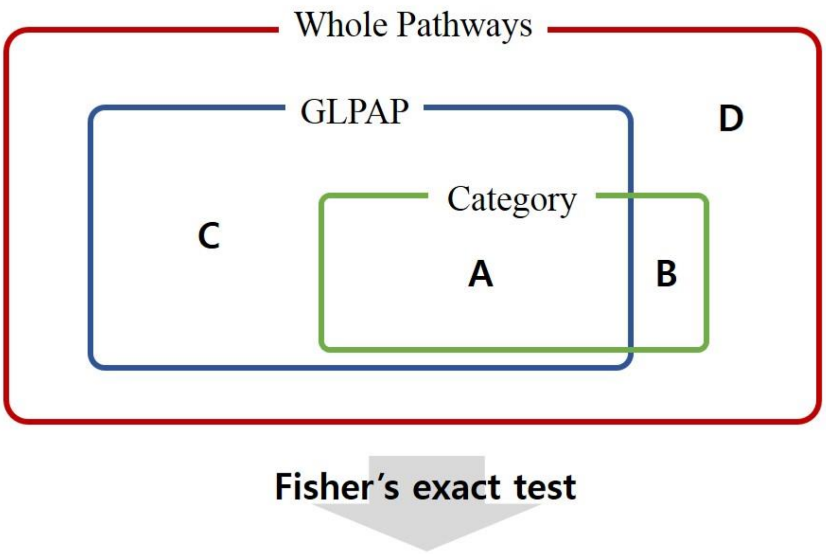

	GLPAP	Not GLPAP
In Category	A	B
Not in Category	C	D

Supplementary Figure 3. A contingency table with two variables to calculate p-values of each category. To calculate GLPAP enrichment for each category of pathways, we built a contingency table for each category according to the two variables. One was whether a pathway is GLPAP or not and the other was whether a pathway belongs to the concerned category.
